# Evaluating the efficacy of two body image online writing interventions against a neutral writing control in targeting positive body image and distress in female cancer survivors: a randomised controlled trial

**DOI:** 10.1007/s00520-026-10732-9

**Published:** 2026-05-09

**Authors:** Elizabeta Brkic, Ivanka Prichard, Lisa Beatty

**Affiliations:** 1 Flinders University Institute for Mental Health & Wellbeing, College of Human Sciences and Culture, Flinders University, Adelaide, SA Australia; 2 Caring Futures Institute, College of Health and Enablement, Flinders University, Adelaide, SA Australia

**Keywords:** Cancer survivors, Females, Body image, Expressive writing, Body functionality appreciation

## Abstract

**Objective:**

This study evaluated the efficacy of two online writing interventions, *Expand Your Horizon (EYH)* and *My Changed Body (MyCB),* compared to control, in improving body image and distress in a cancer population, and investigated whether they target different mechanisms.

**Methods:**

Adult female cancer survivors (*N* = 132) were randomised to *EYH, MyCB,* or a neutral writing control. Body appreciation, body dissatisfaction, body functionality appreciation, self-compassion and distress were measured at baseline, immediately post-intervention, 1-week, and 2-week follow-up.

**Results:**

*EYH* participants demonstrated significantly higher body appreciation (*p* = .002, *d* = 0.31) and significantly lower body dissatisfaction (*p* = .002, *d* = −0.22) immediately post-intervention compared to control. The same pattern existed for *MyCB* participants but was not significant. No significant differences in proposed mechanisms—self-compassion or functionality appreciation—emerged between groups. Main effects of time emerged on body dissatisfaction, distress, and self-compassion at the 1-week and 2-week follow-ups, with all participants improving over time.

**Conclusion:**

The findings support the efficacy of *EYH* to improve state body appreciation and reduce body dissatisfaction in female cancer survivors. Further research is necessary to determine the optimal dose needed to produce sustained improvements in body image and distress.

**Supplementary Information:**

The online version contains supplementary material available at 10.1007/s00520-026-10732-9.

## Introduction

Hair loss, skin discolouration, scarring, and weight fluctuations are some of the appearance-related side effects female cancer survivors may experience following diagnosis and treatment [1]. Side effects may vary according to cancer and treatment type and can significantly affect a woman’s attitude towards and perception of her body, often leading to negative body image [1]. Body dissatisfaction is highly prevalent and can persist for years after cancer diagnosis and treatment [2], particularly among female cancer survivors, who report significantly more body image concerns than male cancer survivors [3]. Additionally, female cancer survivors are at increased risk of depression, anxiety, impaired quality of life (QoL), psychosocial distress, and isolation [2]. Therefore, promoting positive body image is essential in enhancing the psychosocial and physical well-being of this population.

Positive body image is a multidimensional construct which is comprised of core features such as love and respect for one’s body [4]. It is both a malleable state and stable trait construct that can be increased via interventions, and maintained over time [5]. It is also distinct from negative body image, allowing individuals to experience both simultaneously [6]. A key facet of positive body image is body appreciation. This involves accepting one’s body regardless of size or imperfections, attending to the body’s needs, and resisting unrealistic media beauty portrayals [7]. To date, interventions to promote body appreciation have focussed on two mechanistic pathways. The first enhances body appreciation by targeting body *functionality* appreciation. This involves appreciating and respecting everything the body is capable of (e.g., communication, creative activities, self-care, internal processes; Alleva & Tylka, 2021). The second is through promoting self-compassion. Self-compassion involves adopting a kind and understanding attitude towards oneself during times of suffering, using three key components: self-kindness, shared humanity, and mindfulness [8].

In support of pathway one, a study exploring breast cancer survivors (BCSs) positive body image identified functionality appreciation as a key factor in facilitating positive body image experiences post-cancer treatment and diagnosis [2]. Furthermore, Ettridge et al. [9] found better global QoL and emotional and cognitive functioning in female BCSs who appreciated their body, and its functionality, compared to women who had negative body image [9]. In support of pathway two, self-compassion may serve a protective role against the negative impacts associated with body dissatisfaction [10]. Self-compassion has been associated with greater psychological wellbeing and body appreciation in female cancer populations [11, 12]. Therefore, interventions which foster either functionality appreciation or self-compassion may provide protection against negative body image among female cancer survivors.

Online writing interventions for targeting body image mechanisms in cancer populations have gained interest in recent years for being low-cost and time-efficient [13]. Writing about past experiences and major life changes, such as a cancer diagnosis, has been associated with benefits including lower stress, increased confidence and emotional regulation [13]. To date, two writing interventions have shown promise: The first, *Expand Your Horizon* (*EYH),* is an online writing intervention targeting functionality appreciation via three 15-min-based writing exercises, wherein participants reflect on and write about the value of different body functions [14]. Evidence from the general population suggests *EYH* is effective in improving trait body appreciation and trait functionality appreciation at 1-week follow-up in comparison to controls [14–16], with a single session variation of *EYH* also demonstrating efficacy [16, 17]. Single-session interventions have gained interest amongst researchers for their potential to deliver rapid benefits and provide a more focused treatment approach, thereby improving engagement [6]. Recently, Brkic et al. [18] explored multi and single-session versions of *EYH* compared to neutral writing controls in female cancer survivors. Results demonstrated a lack of feasibility for the multi-session format with severe attrition observed [18]. While the single-session version demonstrated feasibility and improved state body image immediately post-intervention, there were no significant differences between *EYH* and control groups on body image or distress [18]. The study’s underpowered design and participants’ inconsistent adherence to intervention instructions impacted intervention effectiveness [18]. Although writing compliance had been achieved in *EYH* studies among the general population [14–16], a more structured writing exercise may be needed for cancer populations.

The second intervention, *My Changed Body* (*MyCB),* uses a 30-min self-compassion-based writing exercise to target body appreciation [1, 12]. *MyCB* was created for use in cancer populations to promote self-compassionate attitudes, enabling them to perceive their cancer experience in a supportive way [12]. Findings revealed sustained improvements in trait body dissatisfaction and trait body appreciation at 1-week, 1-month and 2-month follow-up in BCSs who completed *MyCB,* relative to control [12]. Furthermore, Przezdziecki and Sherman [1] showed that participants using self-compassionate prompts exhibited reduced negative emotions when recalling a negative body image event, compared to unstructured writing.

Both *EYH* and *MyCB* show promise for enhancing body appreciation, but through distinct mechanisms — body functionality appreciation for *EYH* and self-compassion for *MyCB*. Although evidence from a systematic review suggests *EYH* is more effective than self-compassion and CBT-based interventions in targeting positive body image among general populations [19], to our knowledge, no existing studies have directly compared *EYH* and *MyCB*, or investigated whether the interventions operate through different mechanisms for a cancer population.

The current study aimed to compare the efficacy of *EYH* and *MyCB* in improving state and trait body image and distress relative to an active control in female cancer survivors. We hypothesised that both interventions would significantly improve body appreciation, body dissatisfaction, and distress immediately post-intervention, and at 1-week and 2-week follow-up compared to control. A secondary aim was to explore whether the interventions operate through different mechanisms. It was hypothesised that *EYH* participants would report significantly higher functionality appreciation at all follow-ups compared to *MyCB* and control; while *MyCB* participants would report significantly higher self-compassion than *EYH* and control.

## Method

### Participants

Participants were recruited online through Australian cancer organisations (e.g., Breast Cancer Network Australia (BCNA)), sponsored social media advertisements, social media cancer support groups, and the online research platform, Prolific (limited to Australia, United Kingdom and New Zealand; [20]). Adult women were eligible if they had an existing or previous cancer diagnosis, were undergoing or had completed active cancer treatment, were proficient in English, and had internet access and a working email address. Data collection occurred from September 2023 to May 2024.

### Procedure

Women participated online via Qualtrics. They provided informed consent and completed baseline measures. Qualtrics then randomly assigned participants to *EYH, MyCB* or control. After writing completion, participants completed post-writing state measures, with follow-up measures completed at 1-week and 2-week post-baseline. The study was approved by the University’s Human Research Ethics Committee in accordance with the National Statement on Ethical Conduct in Human Research (No. 4372), and pre-registered through the Australian and New Zealand Clinical Trials Registry (Trial No. ACTRN12623000739617p; date registered: 07 July 2023).

### Interventions

Participants in all groups completed one single structured-writing session, and were instructed to (1) spend up to 30 min writing, (2) write as much as they could in that time, and (3) attempt to answer all the writing prompts once they had started writing.

#### Expand your horizon

The single-session EYH format was selected due to demonstrating feasibility in a cancer population [18]. Participants were introduced to the concept of body functionality and given a list containing six categories of possible body functions which they could refer to throughout the session (i.e., *sensations; physical activity; health; creative endeavours; self-care* and *communication*). Participants were instructed to reflect on the meaning and importance they associated with each function.

Two modifications were made to the *EYH* intervention: First, the writing duration was increased to 30 min to ensure consistency in length across the *EYH* and *MyCB* writing conditions. Second, the writing task was divided into six sections with structured prompts aligned to the original three session *EYH* intervention [14], with each prompt focusing on a different category of body functions.

#### My changed body

*MyCB* participants first recalled and wrote about a distressing event following their cancer diagnosis [12]. Next, participants were guided by five self-compassion-based prompts to (1) focus on how their body had changed since this cancer diagnosis; (2) show kindness to their experience the same way they would to a friend in a similar situation using a broad self-compassionate perspective; (3) write about ways in which other female cancer survivors share similar experiences; (4) examine their feelings using a bigger perspective by putting space between the event and their reaction; and (5) write a self-compassionate letter to themselves.

#### Active control

Active control participants were asked to write about different rooms in their house as though they were describing it to someone who had never seen it before [21]. Participants were guided by six writing prompts which asked them to provide detailed information on each room (e.g., size).

### Measures

The following battery of psychometrically validated measures was administered at baseline, immediately post-intervention (state measures), and at 1-week and 2-week follow-up (trait measures).

#### Demographic and clinical information

At baseline, participants reported their age, sex, ethnicity, educational attainment, relationship status, income, cancer type, date of diagnosis, cancer stage, treatment(s) received, and status of cancer treatment.

#### State body image

Visual analogue scales (VAS) were used to measure state body appreciation, functionality appreciation, and body dissatisfaction immediately before and after writing. Participants rated how they felt in the present moment on a scale from 0 (*not at all*) to 100 (*very much*) on a 100-mm horizontal line [18].

State body appreciation (primary outcome) was assessed using a state version of the 2-item short form trait Body Appreciation Scale-2 (BAS-2SF) [22]. The BAS-2SF is a measure of positive body image which assesses an individual’s acceptance, respect, and/or appreciation for their body [23]. Scores for the two items were averaged. Higher scores reflected greater body appreciation ($\alpha$ = 0.90). Secondary outcomes included state functionality appreciation, assessed using selected items from the Functionality Appreciation Scale (FAS; [24]), state self-compassion, measured with the State Self-Compassion Scale–Short Form (SSCS-SF; [22]), body dissatisfaction, evaluated using the Body Image States Scale (BISS; [25]), and psychological distress, assessed with the Distress Thermometer (NCCN; 1999; see Table 1).

**Table 1:** Measures of trait and state body image and distress secondary outcomes

Domain/measure	Description
*State body image and distress*	
Functionality Appreciation; The Functionality Appreciation Scale (FAS; [24])	State functionality appreciation was measured using two VAS items from the 7-item trait FAS as per Brkic et al. ([18], [24]) with minor wording adjustments for state assessment. Items were averaged, with higher scores indicating higher functionality appreciation ($\alpha$ = .93)
Self-Compassion; 6-item State Self-Compassion Scale-Short Form (SSCS-SF; [22])	The SSCS-SF utilises a 5-point scale (0 = *not at all true for me* to 5 = *very true for me*; [22]). The introductory statement was modified to prompt participants to reflect on their cancer diagnosis rather than a painful or difficult event. Negatively worded items were reverse scored, and all items were averaged to obtain a total score, with higher scores reflecting greater self-compassion (score range, 0 to 5, $\alpha$ = .78)
Body Dissatisfaction; Body Image States Scale (BISS; [25])	In line with Brkic et al. [18] three of the six items from the BISS were selected to obtain a measure of state body dissatisfaction, which measure an individual’s current perception about their physical appearance [18]. The scale was changed from a Likert scale to a VAS to ensure consistency across state measures. Items were reverse scored and averaged; with higher scores indicating higher body dissatisfaction ($\alpha$ = .93)
Distress; Distress Thermometer [26]	Participants reported their level of distress in the present moment on a 10-point scale (0 = *no distress* to 10 = *extreme distress*)
*Trait body image and distress*	
Functionality Appreciation; The Functionality Appreciation Scale (FAS; [24])	The FAS contains seven items rated on a five-point scale, with options ranging from 1 = *strongly disagree,* to 5 = *strongly agree.* Participants’ scores on the FAS were averaged; higher scores indicated higher functionality appreciation (score range; 1 to 5; $\alpha$ = .93)
Self-Compassion; The 12-item Self-Compassion Scale-Short Form (SCS-SF; [27])	The SCS-SF utilises a 5-point response scale (1 = *almost never,* 5 = *almost always*; [27]). All negatively worded items were reverse scored. A total score was obtained by averaging the 12-items,with higher scores indicating higher self-compassion (score range, 1 to 5; $\alpha$ = .88)
Body Dissatisfaction; Body Image Scale (BIS; [28])	The 10-item BIS was used to assess body dissatisfaction [28]. Items are rated on a 4-point scale from 0 = *not at all* to 3 = *very much.* Scores were summed; higher scores reflected higher body image disturbance (score range; 0 to 30; $\alpha$ = .92)
Distress; 10-item Depression, Anxiety, Stress Scales (DASS-10; [29])	The DASS-10 utilises a 4-point scale (0 = *did not apply to me at all* to 3 = *applied to me very much or most of the time*; [29]). A single distress score was obtained by averaging the items,higher scores indicated higher distress (score range, 0 to 3; $\alpha$ = .88)

#### Trait measures

Trait body appreciation (primary outcome) was assessed using the trait version of the two-item Body Appreciation Scale-2 Short Form (BAS-2SF; [23]). Items were rated from 1 = *never* to 5 = *always.* Scores on the two items were averaged with higher scores indicating greater body appreciation (range = 1 to 5; $\alpha$ = 0.85). Secondary outcomes included trait functionality appreciation, assessed using the FAS [24], self-compassion, measured with the 12-item Self-Compassion Scale–Short Form (SCS-SF; [27]), body dissatisfaction, evaluated using the Body Image Scale (BIS; [28]), and psychological distress, assessed with the Depression, Anxiety, Stress Scales–10 item version (DASS-10; [29], see Table 1).

### Statistical analyses

Sample size calculations in G*Power (version 3.1.9.7.) indicated that 78 participants (26 participants per group) were needed, assuming a Cohen’s *d* effect size of 0.5 [12, 30], 80% power, and a significance level of 0.05. Accounting for an anticipated dropout rate of 20%, we aimed to recruit 99 participants.

Analyses were conducted in IBM SPSS Statistics (Version 28; [31]). One-way ANOVAs, independent samples *t*-tests, and chi-square tests were used to examine baseline sociodemographic and clinical differences between: (i) intervention groups, (ii) study completers versus dropouts and (iii) participants recruited through Prolific versus other avenues. Intervention effects were explored using linear mixed models (LMM) based on a group by time randomised design with 95% confidence intervals. LMM handle data that is missing at random and account for attrition, as they only require participants to have data from at least one time point. An unstructured covariance matrix was utilised, and baseline data was included as an observation, with one exception where baseline was included as a covariate to control for baseline differences.

Main effects of group and time were not reported in the presence of significant interactions. Unadjusted and adjusted models were used to control for baseline group differences on sociodemographic and clinical variables, and recruitment sourcing that emerged. As the pattern did not differ, only adjusted model results are presented. Between-group and within-group effects sizes were calculated using Cohen’s *d,* with 0.20, 0.50, and 0.80 classified as small, moderate, and large, respectively [32].

## Results

### Participants

**Fig. 1:**
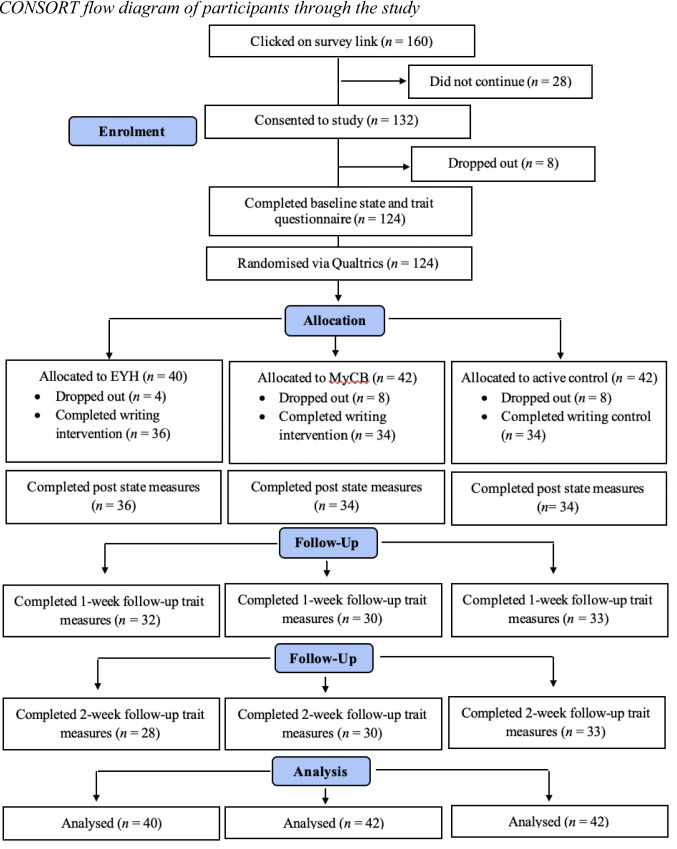
CONSORT flow diagram of participants through the study. *Note.* All participants with data from at least one time point were included in the analyses, as linear mixed model analyses account for attrition within the model

Figure 1 summarises participant flow through the study. Overall, 132 participants consented to participate. Of these, 8 (6.06%) dropped out prior to randomisation (during the baseline questionnaire), resulting in 124 participants included in the LMM analyses. A further 20 (15.15%) dropped out following randomisation, with 104 participants completing the writing task. Sociodemographic, clinical, and psychosocial characteristics of the sample at baseline are summarised in Table 2. The groups did not significantly differ across any characteristic.

**Table 2:** Baseline sociodemographic and clinical characteristics of sample

Baseline characteristic	EYH (*n* = 40)	MyCB (*n* = 42)	Control (*n* = 42)	Sig	Effect size
	Mean (*SD)*	Mean (*SD)*	Mean (*SD)*	*p*	$$\eta^2$$
Age	54.85 (11.87)	54.97 (11.58)	56.83 (12.69)	.70	.00
Years since diagnosis	11.15 (9.40)	9.40 (7.61)	9.64 (7.62)	.58	.01
	*n*(%)	*n*(%)	*n*(%)	*p*	$$\varphi_c$$
*Education*				.15$$\dagger$$	.15
Secondary	2 (5.0)	7 (16.7)	8 (19.0)		
Tertiary	36 (90.0)	35 (83.3)	33 (78.6)		
*Ethnicity*				.91$$\dagger$$	.06
Caucasian/White	37 (92.5)	49 (95.2)	39 (92.9)		
*Sourcing*				.70$$\dagger$$	.19
Flinders University	0	5 (11.9)	2 (4.9)		
Cancer Council	3 (7.5)	1 (2.4)	1 (2.4)		
BCNA	10 (25.0)	8 (19.0)	8 (19.5)		
Prolific	22 (55.0)	22 (52.4)	21 (51.2)		
Support group	2 (5.0)	2 (4.8)	3 (7.3)		
Researcher contact	0	1 (2.4)	1 (2.4)		
Other^a^	3 (7.5)	3 (7.1)	5 (12.2)		
*Relationship status*				.41	.12$$\ddagger$$$$\ddagger$$
Married/partnered	19 (47.5)	24 (57.1)	18 (42.9)		
*Annual gross income*				.41	.13
<$45,000	14 (35.0)	15 (35.7)	22 (52.4)		
>$45,000	20 (50.0)	19 (45.2)	16 (38.1)		
Prefer not to say	6 (15.0)	8 (19.0)	4 (9.5)		
*Cancer type*					
Breast	25 (62.5)	30 (71.4)	24 (57.1)	.61$$\dagger$$	.16
Other^b^	15 (37.5)	12 (28.6)	18 (42.9)	.38	.12
*Cancer Stage*				.15	.16
Early stage (0–3)	30 (75.5)	25 (59.5)	26 (61.9)		
Metastatic	2 (5.0)	5 (11.9)	9 (21.4)		
Unsure	8 (20.0)	12 (28.6)	7 (16.7)		
*Treatment received*^c^					
Surgery	35 (87.5)	39 (92.9)	34 (81.0)	.27	.15
Chemotherapy	24 (60.0)	25 (59.5)	20 (47.6)	.44	.12
Radiotherapy	29 (72.5)	27 (64.3)	22 (52.4)	.16	.17
Endocrine therapy	13 (32.5)	19 (45.2)	13 (31.0)	.33	.13
Immunotherapy	4 (10.0)	2 (4.8)	3 (7.1)	.63$$\dagger$$	.08
Other^d^	7 (17.5)	1 (2.4)	6 (14.3)	.07$$\dagger$$	.21
*Completed treatment*				.63$$\dagger$$	.11
Yes	31 (77.5)	30 (71.4)	29 (69.0)		

The total number of participants represents participants who provided complete baseline data prior to randomisation

^a^Email (*n* = 2), social media (*n* = 1), Facebook (*n* = 3), Friend (*n* = 1)

^b^Bowel (*n* = 7), melanoma (*n* = 6), brain (*n* = 1), skin (*n* = 2), vaginal (*n* = 3), thyroid (*n* = 6), anal (*n* = 1), cervical (*n* = 6), kidney (*n* = 2), ovarian (*n* = 8), lymphoma (*n* = 3), bone (*n* = 1), leiomyosarcoma (*n* = 1), basal cell cancer (*n* = 1), colon (*n* = 1), blood cancer (*n* = 1)

^c^Multiple responses allowed

^d^Brachia (*n* = 1), Herceptin (*n* = 4), CDK inhibitor (*n* = 1), Nephrectomy (*n* = 1), Radioactive Iodine therapy (*n* = 1), MOHS surgery (*n* = 1), CyberKnife (*n* = 1), Stereotactic body radiation therapy (*n* = 1), Hysterectomy (*n* = 1), Ovariectomy (*n* = 1), cream (*n* = 1)

$\dagger$Fisher’s Exact Test was reported for this variable due to <20% of cells having an expected frequency <5

$\ddagger$Phi Coefficient was reported for this variable

Table 3 presents baseline scores in outcome variables across groups. There was a statistically significant difference in trait body appreciation scores between *EYH* and *MyCB* participants, whereby *EYH* participants had greater baseline body appreciation scores than *MyCB* participants, *F* (2, 121) = 3.11, *p* = 0.04, ${\eta }^{2}$ = 0.5. To account for this difference, baseline body appreciation was included as a covariate in the body appreciation analysis.

**Table 3:** Baseline descriptive statistics and one-way ANOVAS on state and trait outcome variables between groups

Outcome variable	EYH (*n* = 40)		MyCB (*n* = 42)		Control (*n* = 42)				
	M (SD)	95 CI%	M (SD)	95 CI%	M (SD)	95 CI%	*F*(*df*)	*p*	${\eta }^{2}$ $\eta^2$
*State variable*									
Functionality appreciation	69.41 (27.52)	60.61, 78.21	64.15 (26.30)	55.74, 72.56	68.48 (24.22)	60.83, 76.12	.46 (2, 118)	.63	.01
Body appreciation	57.80 (27.38)	49.04, 66.56	45.94 (30.01)	36.21, 55.66	46.01 (27.55)	37.42, 54.60	2.34 (2, 118)	.10	.04
Distress	2.33 (2.40)	1.56, 3.11	3.36 (2.84)	2.47, 4.24	3.10 (2.77)	2.23, 3.96	1.58 (2, 120)	.21	.03
Self-compassion	3.37 (.85)	3.09, 3.64	3.37 (.91)	3.09, 3.66	3.50 (.78)	3.25, 3.74	.30 (2, 120)	.74	.00
Body dissatisfaction	54.11 (25.29)	46.02, 62.20	65.05 (28.02)	56.09, 74.01	63.24 (25.11)	55.41, 71.07	2.02 (2, 119)	.14	.03
*Trait variable*									
Functionality appreciation	4.12 (.86)	3.85, 4.40	3.73 (.89)	3.45, 4.01	3.97 (.73)	3.74, 4.08	2.33 (2, 121)	.10	.04
Body appreciation	**3.21 (1.05)**	**2.88, 3.55**	**2.67 (1.05)**	**2.34, 2.99**	2.81 (.97)	2.51, 3.11	3.11 (2, 121)	**.04**	.05
Distress	.75 (.53)	.58,.93	.83 (.56)	.66, 1.01	.88 (.67)	.72,.93	.44 (2, 121)	.65	.01
Self-compassion	2.97 (.79)	2.72, 3.22	3.05 (.80)	2.80, 3.30	2.90 (.72)	2.68, 3.13	.39 (2, 121)	.67	.01
Body dissatisfaction	12.40 (8.02)	9.83, 14.97	14.67 (8.86)	11.90, 17.43	12.88 (8.26)	10.31, 15.46	.84 (2, 121)	.43	.01

*CI *confidence interval

### Recruitment source analysis

Participants recruited through Prolific (*n* = 66) significantly differed to participants recruited through other avenues (*n* = 60) across a number of baseline sociodemographic and clinical variables (Supplementary Table S1), and baseline state and trait outcome variables (Supplementary Table S2). As such, recruitment source was added as a covariate to all analyses.

### Drop-out analysis

Dropouts (*n* = 28) and intervention completers (*n* = 104) differed significantly on recruitment sourcing at baseline (Supplementary Table S3). Participants recruited through Prolific were more likely to complete the study in comparison to participants recruited through other recruitment avenues (${\chi }^{2}$(6) = 44.76, *N* = 128, *p* = < 0.001, *phi* = 0.57). Dropouts and completers also significantly differed on types of cancer treatment received, with more intervention completers receiving radiotherapy. No significant differences between dropouts and completers on baseline outcome variables were found (Supplementary Table S4).

### State outcomes

There was a significant interaction for state body appreciation, *F*(2, 103.08) = 6.75, *p* = 0.002 (see Table 4); *EYH* had significantly higher state body appreciation immediately post-intervention compared to control (*Mdifference* = 19.89, *p* = 0.002*, d* = 0.31)*.* There were no significant differences between *EYH* and *MyCB* (*Mdifference* = 7.99, *p* = 1.00, *d* = −0.14) or *MyCB* and control (*Mdifference* = 11.89, *p* = 0.117, *d* = 0.45).

**Table 4:** Descriptive statistics and group-by-time interaction effects for state and trait outcome variables at baseline and immediately post-intervention

			Baseline	95% CI	Immediately post-intervention		95% CI	Group-by-time interaction
*State outcome variable*		Group	M (SE)		M (SE)			F(*p*)
Functionality appreciation		EYH	69.30 (4.11)	61.17, 77.44	78.93 (3.37)		72.26, 85.60	**5.93 (.004)**
		MyCB	64.28 (4.10)	56.18, 72.38	74.65 (3.38)		67.95, 81.34	
		Control	69.48 (4.11)	61.33, 77.62	69.03 (3.40)		62.29, 75.77	
Body appreciation		EYH	57.78 (4.47)	48.93, 66.63	68.61 (4.01)		60.67, 76.55	**6.75 (.002)**
		MyCB	45.92 (4.50)	37.01, 54.84	60.61 (4.05)		52.59, 68.63	
		Control	46.93 (4.42)	38.17, 55.68	48.72 (4.01)		40.76, 56.66	
Distress		EYH	2.36 (.41)	1.54, 3.18	1.77 (.39)		1.01, 2.57	.33 (.72)
		MyCB	3.23 (.40)	2.43, 4.03	2.44 (.39)		1.66, 3.22	
		Control	3.10 (.41)	2.30, 3.91	2.65 (.40)		1.86, 3.43	
Self-compassion		EYH	3.33 (.14)	3.06, 3.61	3.62 (.12)		3.38, 3.86	**3.28 (.04)**
		MyCB	3.39 (.13)	3.12, 3.65	3.88 (.12)		3.63, 4.12	
		Control	3.50 (.13)	3.24, 3.77	3.65 (.12)		3.41, 3.90	
Body dissatisfaction		EYH	54.06 (4.13)	45.89, 62.23	42.42 (4.12)		34.25, 50.59	**6.78 (.002)**
		MyCB	65.20 (4.11)	57.07, 73.34	46.99 (4.13)		38.80, 55.18	
		Control	62.67 (4.08)	54.59, 70.76	56.93 (4.13)		48.76, 65.11	
		Baseline	95% CI	1-week	95% CI	2-weeks	95% CI	Group-by-time interaction
*Trait outcome variable*	Group	M (SE)		M (SE)		M (SE)		F(*p*)
Functionality appreciation$\parallel$	EYH	4.12 (.13)	3.86, 4.38	4.23 (.12)	3.99, 4.47	4.13 (.13)	3.86, 4.39	.99 (.42)
	MyCB	3.74 (.13)	3.48, 4.00	3.91 (.12)	3.67, 4.15	3.89 (.13)	3.63, 4.15	
	Control	4.00 (.13)	3.74, 4.26	4.01 (.12)	3.77, 4.24	3.93 (.13)	3.67, 4.19	
Body appreciation$\parallel$	EYH	3.20 (.16)	2.88, 3.52	3.36 (.15)	3.07, 3.66	3.41 (.16)	3.09, 3.73	.51 (.73)
	MyCB	2.63 (.16)	2.31, 2.95	2.91 (.15)	2.61, 3.20	2.93 (.16)	2.62, 3.24	
	Control	2.82 (.16)	2.50, 3.13	2.92 (.15)	2.63, 3.21	3.06 (.15)	2.75, 3.36	
Distress$\parallel$	EYH	.75 (.09)	.56,.93	.62 (.11)	.41,.84	.57 (.10)	.37,.78	.87 (.48)
	MyCB	.81 (.09)	.63,.99	.76 (.11)	.55,.97	.78 (.10)	.58,.98	
	Control	.87 (.09)	.68, 1.05	.85 (.11)	.64, 1.06	.78 (.10)	.58,.98	
Self-compassion$\parallel$	EYH	2.97 (.12)	2.72, 3.21	3.07 (.13)	2.80, 3.34	3.13 (.14)	2.85, 3.40	2.10 (.09)
	MyCB	3.05 (.12)	2.81, 3.35	3.09 (.13)	2.82, 3.35	3.10 (.14)	2.82, 3.37	
	Control	2.91 (.12)	2.67, 3.15	3.11 (.13)	2.84, 3.37	3.25 (.14)	2.98, 3.53	
Body dissatisfaction$\parallel$	EYH	12.36 (1.33)	9.71, 15.02	10.87 (1.46)	7.98, 13.76	8.86 (1.41)	6.07, 11.64	1.94 (.11)
	MyCB	14.49 (1.32)	11.88, 17.11	11.31 (1.46)	8.41, 14.20	11.68 (1.39)	8.93, 14.44	
	Control	12.80 (1.33)	10.17, 15.43	11.61 (1.45)	8.74, 14.47	11.01 (1.34)	8.28, 13.74	

$\mathit\parallel$Controlling for recruitment source

*CI *confidence interval

For state body dissatisfaction, there was a significant interaction, *F*(2, 102.18) = 6.78, *p* = 0.002 (see Table 4). *EYH* participants had significantly lower body dissatisfaction over time compared to control (*Mdifference* = −14.51, *p* = 0.043, *d* = −0.22). No significant differences emerged between *EYH* and *MyCB* (*Mdifference* = −4.57, *p* = 1.00, *d* = 0.25) or *MyCB* and control (*Mdifference* = −9.93, *p* = 0.27, *d* = −0.47).

No significant interaction or group main effect was found on state distress (see Table 4). However, there was a significant main effect of time, *F*(1, 102.644) = 12.85, *p* = < 0.001, whereby all groups experienced small improvements over time (*d* range 0.17 to 0.32).

There were significant interactions for state functionality appreciation, *F*(2, 106.44) = 5.93, *p* = 0.004, and state self-compassion, *F*(2, 107.15) = 3.28, *p* = 0.04 (see Table 4). Although post-hoc testing revealed no significant differences between groups, within group effect sizes indicated comparable moderate improvements in functionality appreciation for *EYH* (*d* = 0.41) and *MyCB* (*d* = 0.44), with negligible improvement (*d* = 0.02) in the control group. Similarly, moderate improvements in self-compassion were observed for *MyCB* (*d* = 0.59) and *EYH* (*d* = 0.35) at post-intervention, while the control group had a small effect (*d* = 0.18).

### Trait outcomes

For trait body appreciation, after controlling for baseline differences there was no significant interaction or main effects of time or group.

For trait body dissatisfaction, there was no significant interaction or main effect of group; however, there was a significant main effect of time, *F*(2, 91.79) = 14.93, *p* = < 0.001 (see Table 4). Within group effect sizes demonstrated small to moderate reductions in body dissatisfaction across time for *EYH* (1-week: *d* = 0.17; 2-weeks: *d* = 0.40) and *MyCB* (1-week: *d* = 0.35; 2-weeks: *d* = 0.32), whilst smaller effects were observed for control (1-week: *d* = 0.13; 2-weeks: *d* = 0.20).

For trait distress, there was no significant interaction or main effect of group, but a significant main effect of time emerged, *F*(2, 90.97) = 3.87, *p* = 0.02 (see Table 4). Within groups effect sizes ranged from 0.03 to 0.28 across groups, suggesting that all groups experienced a small reduction in distress over time.

No significant interaction or main effects of time or group were found for trait functionality appreciation from pre-intervention to follow-up (see Table 4).

No significant interaction or group main effect was found for trait self-compassion; however, there was a significant main effect of time, *F*(2, 90.76) = 8.55, *p* < 0.001 (see Table 4). Within-groups effect sizes demonstrated small improvements in trait self-compassion for *EYH* (*d* = 0.12 to 0.19) and *MyCB* (*d* = 0.04 to 0.05) from pre-intervention to 1-week and 2-week follow-up. In comparison, the control group demonstrated a moderate improvement in trait self-compassion, with Cohen’s *d* ranging from 0.24 to 0.42, indicating that the time main effect may have been driven by the control group.

### Exploratory analysis: intervention compliance

Exploratory analyses examined whether compliance to writing instructions influenced within-group differences over time for *EYH* and control participants. Compliance amongst *MyCB* participants was not investigated due to a negligible number of non-compliant individuals. For *EYH,* responses were coded as compliant (*n* = 17) when they focused on functionality appreciation, and non-compliant (*n* = 19) when they focused on body dissatisfaction. Control responses were coded as compliant (*n* = 16) when they wrote about their house in a factual manner; or non-compliant (*n* = 18) when they described their house using emotive language (see Supplementary Tables S5 and S6 for state and trait changes over time by group and compliance).

Overall, *EYH* compliers showed larger improvements in state and trait outcomes, except for distress (*d* = 0.72) and self-compassion (*d* = 0.61), whereby *EYH n*on-compliers improved moderately. Meanwhile, control non-compliers demonstrated small improvements in state outcomes, except for functionality appreciation, where no differences between groups emerged. For trait outcomes, control non-compliers demonstrated moderate improvements in trait body dissatisfaction (*d* = 0.30) and self-compassion at 2 weeks (*d* = 0.60), compared to control compliers (*d* = 0.28).

## General discussion and conclusion

### Discussion

This study compared two online writing interventions, *Expand Your Horizon (EYH)* and *My Changed Body (MyCB),* against an active control, and found that *EYH* participants experienced immediate (state) improvements in body appreciation and body dissatisfaction relative to control, while all three conditions experienced small improvements in state distress. However, there were no trait effects at follow-up. The study also investigated whether the interventions target different mechanisms. As expected, *MyCB* participants demonstrated the largest trends in improvement for state self-compassion at post-intervention. However, contrary to expectations, state functionality appreciation improved similarly for both intervention groups.

### State outcomes

The finding that *EYH* participants reported significantly greater improvements in state body appreciation and body dissatisfaction at post-intervention compared to control extends prior experimental research in both women from the general population [14–16, 33] and cancer survivors [18], and supports prior research demonstrating that targeting body functionality leads to higher levels of body appreciation [14]. Although not statistically significant, *MyCB* was also associated with larger effect sizes for improvements in state body appreciation and body dissatisfaction compared to control. This finding highlights the potential of *MyCB* to target state body image, an area unexplored in previous research [12].

The finding that *MyCB* yielded similar improvements to *EYH* in state functionality appreciation was surprising given that *MyCB* does not target functionality appreciation. However, one study previously found a positive association between functionality appreciation and self-compassion in populations with and without chronic physical conditions [34]. Our results therefore extend this literature by suggesting that female cancer survivors who complete *MyCB* not only develop a self-compassionate perspective but may also enhance their ability to appreciate their body’s functionality. This is an important finding that demonstrates the benefits that *MyCB* can have in improving these body image outcomes.

### Trait outcomes

Contrary to predictions, there were no significant differences between *EYH, MyCB,* or control participants on any trait outcomes. This contradicts previous *MyCB* research, which showed improvements in body dissatisfaction at 1-month and body appreciation at 3-months in BCSs [12].

The finding that *EYH* participants did not demonstrate significant improvements in trait body image or distress is consistent with previous research exploring the single-session version of *EYH* [6]. Whilst some *EYH* studies demonstrated improvements in trait body image in other populations [8, 15–17], the three-session version of *EYH* was utilised*.* Thus, it is not surprising that intervention benefits weakened for *EYH* participants at 1-week and 2-week follow-up given single-session interventions are utilised to provide immediate benefits [6]. More research is needed to inform the optimal timing and dose of writing required to lead to sustained benefits [27].

There were, however, significant main effects of time which revealed improvements in trait distress, body dissatisfaction, and self-compassion across groups. Although unexpected, the control group demonstrated a moderate improvement in trait self-compassion compared to the small improvements found for *EYH* and *MyCB* participants. A potential reason for this relates to compliance with instructions. Building on previous research [18], we sought to minimise non-compliance via replicating the more structured writing instructions from the original *EYH* intervention [14]. Despite this, 19 out of 36 participants still wrote about side effects from their cancer diagnosis. This may suggest that writing about functionality appreciation is challenging for some cancer survivors, especially given the significant psychosocial and physical impacts of body image disturbance in this population (Thornton & Smith, 2023 [3]). Interestingly, *EYH* non-compliers demonstrated moderate improvements in trait distress and self-compassion compared to compliers. Research indicates that writing about traumatic experiences using an emotional expression and cognitive processing approach can facilitate growth and help individuals find meaning [35]. For *EYH* non-compliers, it is possible that writing about body limitations contributed to these improvements by helping them to process bodily changes caused by cancer. Similar to previous findings [18] control non-compliers demonstrated small to moderate improvements in body appreciation, body dissatisfaction, distress, and self-compassion compared to compliers. This was likely driven by the emotive and sentimental ways they wrote about their homes, akin to expressing gratitude. Although gratitude was not directly measured, research shows that individuals who are grateful demonstrate increased self-awareness and acceptance of flaws thereby facilitating self-compassionate attitudes [36].

### Clinical implications

Taken collectively, compliance analyses suggest that *EYH* targets the body image mechanisms it was designed for; however, non-compliers may experience benefits in different mechanisms, such as gratitude. This shows the importance of tailoring writing interventions to meet cancer survivors needs. Future research could explore how writing therapeutically about body limitations caused by cancer (i.e., the trauma emotional expression approach) compares to writing about functionality appreciation. The near-perfect adherence to *MyCB* is also noteworthy, suggesting that *MyCB* may be easier to complete and thus potentially more feasible for female cancer survivors than *EYH.* Although the present findings highlight the short-term potential of *EYH*, it is unlikely to constitute a comprehensive intervention for clinically significant body dissatisfaction when delivered as a stand-alone intervention. Rather, *EYH* may be most effectively conceptualised as one component within a broader psychosocial intervention framework, which may enhance the durability of effects and facilitate longer-term maintenance of gains. Accordingly, future research should examine how *EYH* can be integrated into comprehensive psychosocial treatment models for cancer survivors experiencing body dissatisfaction.

### Limitations

Our findings should be considered in light of potential limitations that future research may wish to explore. First, control participants were not explicitly instructed to write objectively. Thus, future research should ensure control participants are instructed to write without the use of emotive expression to minimise expressive writing. Second, most participants were BCSs which may be the result of high recruitment through BCNA. Therefore, results may not be representative of all cancer survivors. Indeed, body image in cancer populations has primarily been studied with BCSs [37], highlighting the need for future research to explore the efficacy of *EYH* and *MyCB* in under-researched cancer populations. Third, the study lacked sufficient power to conduct subgroup analyses to determine whether benefits varied based on cancer-related factors (e.g., cancer type). Finally, the heterogeneity of the sample warrants consideration. Participants included individuals both undergoing active treatment and those who had completed treatment, and were not selected based on the presence of elevated body image concerns at baseline. Consequently, variability in cancer-related experiences and baseline levels of body dissatisfaction may have influenced the magnitude of intervention effects. Although this broad inclusion enhances generalisability across cancer survivors, it may limit conclusions regarding efficacy among individuals experiencing clinically elevated body dissatisfaction. Future research would benefit from evaluating this intervention within more targeted subgroups of cancer survivors, particularly those with elevated body dissatisfaction, to better determine its potential.

### Conclusion

To our knowledge, this is the first study to directly compare *EYH* and *MyCB* in a distinct population of female cancer survivors and to explore whether they target different mechanisms. Findings support the efficacy of *EYH* to increase state body appreciation and reduce state body dissatisfaction in female cancer survivors, although *MyCB* showed nonsignificant trends towards improvement. However, findings suggest that *MyCB* can target functionality appreciation and self-compassion, and may be easier to adhere to than *EYH.* Future research should determine the optimal dose of *EYH* and *MyCB* needed to produce sustained improvements in body image and distress to ensure maximum intervention benefit.

## Supplementary Information

Below is the link to the electronic supplementary material.

ESM 1 (DOCX 37.4 KB)

## Data Availability

The data supporting this study are not publicly available in order to protect the privacy and confidentiality of study participants.
