# Supplementary material for: Evaluating the efficacy of two body image online writing interventions against a neutral writing control in targeting positive body image and distress in female cancer survivors: a randomised controlled trial
Source: Support Care Cancer. 2026 May 9;34(6):525. doi: 10.1007/s00520-026-10732-9 (PMC13157414; doi:10.1007/s00520-026-10732-9)
Supplement: Supplementary file 1 — (DOCX 37.4 KB) [file 520_2026_10732_MOESM1_ESM.docx]

**Supplementary Files**

Significant differences were found in baseline sociodemographic and clinical variables between Prolific participants (*n* = 66) and participants recruited through other avenues (*n* = 60; Supplementary Table S1), including education where a higher proportion of Prolific participants completed secondary and tertiary education, had an annual income above $45,000, had early-stage cancer, and completed active treatment. Prolific participants also had significantly fewer breast cancer diagnoses ($\chi^{2}$(4) = 28.28, *N* = 126, *p* <.001, *phi* = .47) and received less chemotherapy, radiotherapy, and endocrine therapy in comparison to other participants.

Significant differences between groups also emerged in baseline state and trait outcome variables (Supplementary Table S2). Prolific participants reported significantly higher state body dissatisfaction and lower trait body appreciation and trait self-compassion than participants recruited through other avenues.

| Baseline characteristic | Prolific  (*n=*66) | Other  (*n=*60) | Sig | Effect size |
| --- | --- | --- | --- | --- |
|  | Mean (*SD)* | Mean (*SD)* | *p* | *d* |
| Age | 54.34 (12.68) | 56.68 (10.76) | .26 | .20 |
| Years since diagnosis | 11.33 (8.31) | 8.60 (7.91) | .06 | .34 |
|  | *n*(%) | *n*(%) | *p* | $\phi$ |
| Education |  |  | .05$\dagger$ | .21 |
| Secondary | 12 (18.2) | 5 (8.3) |  |  |
| Tertiary | 54 (81.8) | 52 (86.7) |  |  |
| Ethnicity |  |  | 1.0$\dagger$ | .01 |
| Caucasian/White | 62 (93.9) | 56 (93.3) |  |  |
| Relationship status |  |  | .87 | -.02 |
| Married/partnered | 32 (48.5) | 30 (50.0) |  |  |
| Annual gross income |  |  | .01 | .27 |
| <$45,000 | 33 (50.0) | 19 (31.7*)* |  |  |
| >$45,000 | 29 (43.9) | 27 (45.0) |  |  |
| Prefer not to say | 4 (6.1) | 14 (23.3) |  |  |
| Cancer type |  |  |  |  |
| Breast | 29 (43.9) | 52 (86.7) | <.001$\dagger$ | .47 |
| Other^a^ | 37 (56.1) | 8 (13.3) | <.001 | -.44 |
| Cancer Stage |  |  | <.001 | .36 |
| Early stage (0.3) | 46 (69.7) | 36 (60.0) |  |  |
| Metastatic | 1 (1.5) | 15 (25.0) |  |  |

**Supplementary Table S1**

*Baseline sociodemographic and clinical characteristics of Prolific versus other participants*

**Table S1.** *Continued*

| Baseline characteristic | Prolific  (*n=*66) | Other  (*n=*60) | Sig | Effect size |
| --- | --- | --- | --- | --- |
|  | *n*(%) | *n*(%) | *p* | $\phi$ |
| Cancer Stage |  |  | <.001 | .36 |
| Unsure | 19 (28.8) | 9 (15.0) |  |  |
| Treatment received^b^ |  |  |  |  |
| Surgery | 56 (84.8) | 54 (90.0) | .39 | -.08 |
| Chemotherapy | 27 (40.9) | 43 (71.7) | <.001 | -.31 |
| Radiotherapy | 31 (47.0) | 49 (81.0) | <.001 | -.36 |
| Endocrine therapy | 13 (19.7) | 33 (55.0) | <.001 | -.36 |
| Immunotherapy | 5 (7.6) | 4 (6.7) | 1.00$\dagger$ | .02 |
| Other^c^ | 8 (12.1) | 6 (10.0) | .70 | .03 |
| Completed treatment |  |  | <.001$\dagger$ | .43 |
| Yes | 59 (89.4) | 32 (53.2) |  |  |

*Note.* The total number of participants represents participants who provided complete baseline data prior to randomisation.

^a^ Bowel (*n* = 7), melanoma (*n* = 6), brain (*n* = 1), skin (*n* = 2), vaginal (*n* = 3), thyroid (*n* = 6), anal (*n* = 1), cervical (*n* = 6), kidney (*n* = 2), ovarian (*n* = 8), lymphoma (*n* = 3), bone (*n* = 1), leiomyosarcoma (*n* =1), basal cell cancer (*n* = 1), colon (*n* = 1), blood cancer (*n* = 1)

^b^ Multiple response allowed.

^c^ Brachia (*n* = 1), Herceptin (*n* = 4), CDK inhibitor (*n* = 1), Nephrectomy (*n* = 1), Radioactive Iodine therapy (*n* = 1), MOHS surgery (*n* = 1), Stereotactic body radiation therapy (*n* = 1), CyberKnife (*n* = 1), Hysterectomy (*n* = 1), Ovariectomy (*n* = 1), cream (*n* = 1).

$\dagger$Fisher’s Exact Test was reported for this variable due to <20% of cells having an expected frequency <5.

**Supplementary Table S2**

*Baseline descriptive statistics and independent samples t-tests on state and trait outcome variables between Prolific participants and other participants*

|  | Prolific (*n* = 66) | |  | Other (*n* = 60) | |  |  |  |  |  |
| --- | --- | --- | --- | --- | --- | --- | --- | --- | --- | --- |
| Outcome variable | M | SD |  | M | SD | 95% CI | *t* | *df* | *p* | Cohen’s *d* |
| State variable |  |  |  |  |  |  |  |  |  |  |
| Functionality appreciation | 68.38 | 26.07 |  | 66.13 | 25.93 | -7.15, 11.65 | .47 | 119 | .64 | .09 |
| Body appreciation | 47.14 | 26.0 |  | 53.16 | 31.53 | -16.55, 4.5 | -1.13 | 104.37 | .26 | .21 |
| Distress | 2.52 | 2.56 |  | 3.41 | 2.79 | -1.84, .06 | -1.85 | 121 | .07 | .33 |
| Self-compassion | 3.32 | .86 |  | 3.52 | .81 | -.50, .10 | -1.33 | 121 | .19 | .24 |
| Body dissatisfaction | **65.72** | **25.17** |  | **55.08** | **26.83** | **1.31, 19.97** | **2.26** | **120** | **.03** | **.41** |
| Trait variable |  |  |  |  |  |  |  |  |  |  |
| Functionality appreciation | 4.01 | .72 |  | 3.86 | .94 | -.14, .44 | 1.02 | 111.76 | .31 | .18 |
| Body appreciation | **2.70** | **.95** |  | **3.13** | **1.09** | **-.78, -.08** | **-2.43** | **130** | **.02** | **.42** |
| Distress | .83 | .57 |  | .81 | .61 | -.18, .23 | .21 | 127 | .83 | .03 |
| Self-compassion | **2.84** | **.69** |  | **3.13** | **.81** | **-.55, -.02** | **-2.15** | **127** | **.03** | **.38** |
| Body dissatisfaction | 13.01 | 8.12 |  | 13.53 | 8.59 | -3.45, 2.42 | -.35 | 125 | .73 | .06 |

*Note.* CI = confidence interval.

**Supplementary Table S3**

| Baseline characteristic | Drop-outs  (*n=* 28) | Completers  (*n=* 104) | Sig | Effect size |
| --- | --- | --- | --- | --- |
|  | Mean (*SD)* | Mean (*SD)* | *p* | *d* |
| Age | 52.89 (7.82) | 56.15 (12.62) | .09 | 0.31 |
| Years since diagnosis | 7.95 (7.66) | 10.47 (8.28) | .19 | 0.32 |
|  | *n*(%) | *n*(%) | *p* | $\phi$ |
| Education |  |  | .11$\dagger$ | .21 |
| Secondary | 2 (9.1) | 15 (14.4) |  |  |
| Tertiary | 18 (81.8) | 88 (84.6) |  |  |
| Ethnicity |  |  | .42$\dagger$ | .05 |
| Caucasian/White | 20 (90.9) | 98 (94.2) |  |  |
| Sourcing |  |  | <.001$\dagger$ | .57 |
| Flinders University | 4 (16.7) | 4 (3.8) |  |  |
| Cancer Council | 1 (4.2) | 4 (3.8) |  |  |
| BCNA | 8 (33.3) | 21 (20.2) |  |  |
| Prolific | 0 | 65 (62.5) |  |  |
| Support group | 4 (16.7) | 3 (2.9) |  |  |
| Researcher contact | 0 | 2 (1.9) |  |  |
| Other^a^ | 7 (29.2) | 5 (4.8) |  |  |
| Relationship status |  |  | .70 | -.03 |
| Married/partnered | 10 (45.5) | 52 (50.0) |  |  |

*Baseline sociodemographic and clinical characteristics of drop-outs versus intervention completers*

**Table S3.** *Continued*

| Baseline characteristic | Drop-outs  (*n=* 28) | Completers  (*n=* 104) | Sig | Effect size |
| --- | --- | --- | --- | --- |
|  | *n*(%) | *n*(%) | *p* | $\phi$ |
| Annual gross income |  |  | .11$\dagger$ | .18 |
| <$45,000 | 5 (22.7) | 47 (45.2) |  |  |
| >$45,000 | 12 (54.2) | 44 (42.3) |  |  |
| Prefer not to say | 5 (22.7) | 13 (12.5) |  |  |
| Cancer type |  |  |  |  |
| Breast | 17 (77.3) | 64 (61.5) | .41$\dagger$ | .18 |
| Other^b^ | 5 (22.7) | 40 (38.5) | .16 | .12 |
| Cancer Stage |  |  | .14$\dagger$ | .18 |
| Early stage (0.3) | 15 (68.2) | 67 (64.4) |  |  |
| Metastatic | 5 (22.7) | 11 (10.6) |  |  |
| Unsure | 2 (9.1) | 26 (25.0) |  |  |
| Treatment received^c^ |  |  |  |  |
| Surgery | 19 (86.4) | 91 (87.5) | 1.00$\dagger$ | -.01 |
| Chemotherapy | 15 (68.2) | 55 (52.9) | .19 | .12 |
| Radiotherapy | 18 (81.8) | 62 (59.6) | .05 | .17 |
| Endocrine therapy | 12 (54.2) | 34 (32.7) | .05 | .17 |
| Immunotherapy | 3 (13.6) | 6 (5.8) | .19$\dagger$ | .12 |
| Other^d^ | 1 (4.5) | 13 (12.5) | .46 | -.09 |
| Completed treatment |  |  | .07$\dagger$ | .18 |
| Yes | 12 (54.2) | 79 (76.0) |  |  |

*Note.* ^a^ Email (*n* = 2), social media (*n* = 1), Facebook (*n* = 3), Friend (*n* = 1).

^b^ Bowel (*n* = 7), melanoma (*n* = 6), brain (*n* = 1), skin (*n* = 2), vaginal (*n* = 3), thyroid (*n* = 6), anal (*n* = 1), cervical (*n* = 6), kidney (*n* = 2), ovarian (*n* = 8), lymphoma (*n* = 3), bone (*n* = 1), leiomyosarcoma (*n* =1), basal cell cancer (*n* = 1), colon (*n* = 1), blood cancer (*n* = 1)

^c^ Multiple responses allowed.

^d^ Brachia (*n* = 1), Herceptin (*n* = 4), CDK inhibitor (*n* = 1), Nephrectomy (*n* = 1), Radioactive Iodine therapy (*n* = 1), MOHS surgery (*n* = 1), Stereotactic body radiation therapy (*n* = 1), Cyberknife (*n* = 1), Hysterectomy (*n* = 1), Ovariectomy (*n* = 1), cream (*n* = 1).

$\dagger$ Fisher’s Exact Test was reported for this variable due to <20% of cells having an expected frequency <5.

**Supplementary Table S4**

*Baseline descriptive statistics and independent samples t-tests on state and trait outcome variables between drop-outs and completers*

|  | Drop-outs (*n* = 28) | |  | Completers (*n* = 104) | |  |  |  |  |  |
| --- | --- | --- | --- | --- | --- | --- | --- | --- | --- | --- |
| Outcome variable | M | SD |  | M | SD | 95% CI | *t* | *df* | *p* | Cohen’s *d* |
| State variable |  |  |  |  |  |  |  |  |  |  |
| Functionality appreciation | 57.36 | 27.93 |  | 69.10 | 25.30 | -11.74, 6.56 | -1.79 | 119 | .08 | 0.44 |
| Body appreciation | 40.25 | 34.75 |  | 51.57 | 27.26 | -29.27, 6.63 | -1.31 | 20.81 | .20 | 0.36 |
| Distress | 3.35 | 3.08 |  | 2.86 | 2.62 | -.82, 1.79 | .74 | 121 | .51 | 0.17 |
| Self-compassion | 3.34 | .82 |  | 3.43 | .85 | -.49, .32 | -.41 | 121 | .68 | 0.11 |
| Body dissatisfaction | 65.95 | 29.55 |  | 59.90 | 25.80 | -6.99, 19.10 | .92 | 120 | .36 | 0.22 |
| Trait variable |  |  |  |  |  |  |  |  |  |  |
| Functionality appreciation | 3.71 | .99 |  | 4.00 | .77 | -.71, .07 | -1.35 | 32.91 | .18 | 0.33 |
| Body appreciation | 2.86 | 1.13 |  | 2.92 | 1.02 | -.51, .34 | -.29 | 130 | .77 | 0.06 |
| Distress | .94 | .64 |  | .79 | .57 | -.11, .40 | 1.13 | 127 | .26 | 0.25 |
| Self-compassion | 2.92 | .75 |  | 2.99 | .77 | -.40, .27 | -.38 | 127 | .70 | 0.09 |
| Body dissatisfaction | 15.35 | 9.81 |  | 12.80 | 7.93 | -1.93, 7.03 | 1.16 | 28.68 | .25 | 0.29 |

*Note.* CI = confidence interval.

**Supplementary Table S5**

*Means (and Standard Deviations) for State Outcome Variables by Compliance to Intervention over Time for EYH, MyCB, and Control*

|  |  | EYH | |  | Control | |  |
| --- | --- | --- | --- | --- | --- | --- | --- |
| State outcome variable | Compliant | Time 1 | Time 2 | Cohen’s *d* | Time 1 | Time 2 | Cohen’s *d* |
| Functionality appreciation | Yes | 68.24 (27.33) | 80.65 (20.53) | 0.51 | 69.06 (26.23) | 69.06 (24.51) | 0.03 |
|  | No | 70.18 (27.24) | 77.74 (24.72) | 0.29 | 73.44 (22.12) | 71.47 (17.35) | 0.01 |
| Body appreciation | Yes | 52.68 (27.35) | 73.68 (24.91) | 0.80 | 46.13 (28.29) | 45.16 (27.20) | 0.03 |
|  | No | 61.11 (25.76) | 63.18 (26.28) | 0.08 | 50.28 (25.46) | 53.46 (19.68) | 0.16 |
| Distress | Yes | 2.76 (2.70) | 1.47 (1.97) | 0.54 | 2.81 (2.66) | 2.56 (2.78) | 0.09 |
|  | No | 1.89 (2.08) | 1.95 (2.91) | 0.02 | 3.39 (2.97) | 2.72 (2.61) | 0.24 |
| Self-compassion | Yes | 3.43 (.94) | 3.99 (.61) | 0.71 | 3.39 (.80) | 3.51 (.74) | 0.15 |
|  | No | 3.33 (.82) | 3.32 (.85) | 0.01 | 3.60 (.74) | 3.78 (.83) | 0.23 |
| Body dissatisfaction | Yes | 58.27 (26.49) | 40.16 (28.17) | 0.66 | 65.58 (26.31) | 61.33 (27.43) | 0.16 |
|  | No | 52.44 (23.73) | 46.32 (23.72) | 0.26 | 58.81 (22.89) | 51.96 (22.69) | 0.30 |

*Note.* Time 1 = pre-intervention. Time 2 = immediately post-intervention.

**Supplementary Table S6**

*Means (and Standard Deviations) for Trait Outcome Variables by Compliance to Intervention over Time for EYH, MyCB, and Control*

|  |  | EYH | | |  | Control | | |  |
| --- | --- | --- | --- | --- | --- | --- | --- | --- | --- |
| Trait outcome variable | Compliant | Time 1 | Time 2 | Time 3 | Cohen’s *d*  (T1-T2, T1-T3) | Time  1 | Time  2 | Time  3 | Cohen’s *d*  (T1-T2, T1-T3) |
| Functionality appreciation | Yes | 4.03 (.85) | 4.22 (.83) | 4.17 (.85) | 0.22, 0.16 | 4.00 (.71) | 4.02 (.70) | 3.88 (.80) | 0.03, 0.16 |
|  | No | 4.15 (.84) | 4.15 (.57) | 4.09 (.54) | 0.0, 0.08 | 4.08 (.78) | 4.02 (.57) | 4.00 (.75) | 0.08, 0.10 |
| Body appreciation | Yes | 3.06 (1.12) | 3.23 (.998) | 3.43 (1.03) | 0.16, 0.34 | 2.59 (1.02) | 2.67 (.94) | 2.83 (.88) | 0.08, 0.25 |
|  | No | 3.29 (.97) | 3.32 (.81) | 3.21 (.82) | 0.03, 0.08 | 2.94 (.98) | 3.06 (.86) | 3.17 (.89) | 0.13, 0.24 |
| Distress | Yes | .82 (.63) | .68 (.69) | .72 (.51) | 0.21, 0.17 | .87 (.58) | .91 (.73) | .66 (.68) | 0.06, 0.33 |
|  | No | .65 (.47) | .55 (.41) | .37 (.30) | 0.23, 0.72 | .73 (.59) | .67 (.62) | .74 (.78) | 0.09, 0.01 |
| Self-compassion | Yes | 2.96 (.98) | 3.09 (1.01) | 2.97 (1.09) | 0.13, 0.01 | 2.80 (.65) | 2.92 (.77) | 3.00 (.79) | 0.17, 0.28 |
|  | No | 3.00 (.64) | 3.02 (.60) | 3.38 (.60) | 0.03, 0.61 | 2.96 (.70) | 3.24 (.82) | 3.44 (.88) | 0.37, 0.60 |
| Body dissatisfaction | Yes | 12.88 (7.65) | 9.93 (7.90) | 8.43 (8.43) | 0.37, 0.55 | 13.75 (8.87) | 13.60 (10.37) | 12.40 (9.32) | 0.01, 0.15 |
|  | No | 12.32 (8.28) | 12.41 (9.17) | 9.21 (8.00) | 0.01, 0.38 | 10.56 (6.47) | 8.67 (6.38) | 8.61 (6.45) | 0.29, 0.30 |

*Note.* Time 1 = pre-intervention Time 2= 1-week post-intervention. Time 3 = 2-weeks post-intervention.
